# Exploring Mental Illness Stigma, Help-Seeking Attitudes, and Behaviors on Social Media: A Machine Learning Approach

**DOI:** 10.1192/j.eurpsy.2025.1987

**Published:** 2025-08-26

**Authors:** Y.-J. Lien, H.-P. Feng

**Affiliations:** 1 National Taiwan Normal University, Taipei, Taiwan, Province of China

## Abstract

**Introduction:**

Mental health literacy (MHL) plays a crucial role in promoting help-seeking behavior. However, negative attitudes toward mental illness still pose a substantial barrier. Social media platforms provide a valuable opportunity to explore the relationship between stigma, help-seeking attitudes, and behaviors through the application of natural language processing (NLP) and machine learning (ML) techniques.

**Objectives:**

This study aims to investigate how attitudes toward reducing mental illness stigma and help-seeking influence actual help-seeking behavior among social media users.

**Methods:**

We analyzed 1,506,333 posts from mental health-related Post Text Table (PTT) forums between January 2018 and January 2024. Posts were preprocessed and categorized into three categories: reducing mental illness stigma, help-seeking attitudes, and help-seeking behaviors. Using Bidirectional Encoder Representations from Transformers (BERT) for scoring, the model achieved a precision of 0.81 and accuracy of 0.89. Logistic regression was then applied to assess the predictive value of stigma reduction and help-seeking attitudes on help-seeking behavior.

**Results:**

The study found that for each one-unit increase in score measuring attitudes toward reducing mental illness stigma, he likelihood of help-seeking behavior increased by 1.35 times (95% CI: 1.21–1.50, *p* < 0.001). Similarly, stronger help-seeking attitudes were associated with a 1.41 times higher likelihood of help-seeking behavior (95% CI: 1.16–1.71, *p* < 0.05). Binary logistic regression analysis further demonstrated that users with more pronounced stigma-reducing attitudes and positive help-seeking attitudes were 1.76 times (95% CI: 1.43–2.17, *p* < 0.001) and 2.62 times (95% CI: 1.48–4.65, *p* < 0.05) more likely to engage in help-seeking behavior, respectively.

**Conclusions:**

This study highlights that stronger attitudes toward reducing mental illness stigma, along with more positive help-seeking attitudes, significantly predict help-seeking behavior. By leveraging machine learning and natural language processing, it offers novel insights into how social media discussions influence mental health behaviors, providing a valuable foundation for future interventions aimed at reducing stigma, fostering positive attitudes toward help-seeking, and encouraging actual help-seeking behaviors.

**Disclosure of Interest:**

None Declared

